# Cetuximab is efficient and safe in patients with advanced cutaneous squamous cell carcinoma: a retrospective, multicentre study

**DOI:** 10.18632/oncotarget.27434

**Published:** 2020-01-28

**Authors:** Henri Montaudié, Julien Viotti, Patrick Combemale, Caroline Dutriaux, Nicolas Dupin, Caroline Robert, Laurent Mortier, Régis Kaphan, Anne-Bénédicte Duval-Modeste, Stéphane Dalle, Julie De Quatrebarbes, Andrea Stefan, Florence Brunet-Possenti, Maria Kogay, Alexandra Picard-Gauci, Gilles Poissonnet, Frédéric Peyrade

**Affiliations:** ^1^Department of Dermatology, University Hospital of Nice, Nice, France; ^2^Epidemiology, Biostatistics and Health Data Departments, Antoine Lacassagne Center, Nice, France; ^3^Department of Dermatology, Centre Léon Bérard, Lyon, France; ^4^Department of Dermatology, University Hospital of Bordeaux, Bordeaux, France; ^5^Department of Dermatology, Hôpital Cochin, APHP, Paris, France; ^6^Department of Dermatology, Institut Gustave Roussy, Villejuif, France; ^7^Department of Dermatology, University Hospital Lille, Lille, France; ^8^Medical Oncology Department, Cannes Hospital, Cannes, France; ^9^Department of Dermatology, University Hospital of Rouen, Rouen, France; ^10^Department of Dermatology, ImmuCare, Institut de Cancérologie des Hospices Civils de Lyon, Centre de Recherche en Cancérologie de Lyon, University Hospital of Lyon, Lyon, France; ^11^Department of Dermatology, CHR, Annecy, France; ^12^Department of Dermatology, University Hospital of Caen, Caen, France; ^13^Department of Dermatology, Hôpital Bichat, APHP, Paris, France; ^14^Medical Oncology Department, Antoine Lacassagne Center, Nice, France; ^15^Department of Surgery, Antoine Lacassagne Center, Nice, France

**Keywords:** cutaneous squamous cell carcinoma, cetuximab, epidermal growth factor receptor

## Abstract

There is no standard of care for unresectable cutaneous squamous cell carcinoma (cSCC). Chemotherapy, alone or combined with radiotherapy, is commonly used mostly as palliative treatment; moreover, its poor safety profile limits its use most of the time, especially in elderly patients. Thus, alternative options are needed. Targeted molecular inhibitors, such as the epidermal growth factor receptor inhibitor cetuximab, seem promising, but data are limited. We retrospectively evaluated clinical outcomes of cetuximab as a single agent in this indication. The primary endpoint was the Disease Control Rate (DCR) at 6 weeks according to RECIST criteria. Secondary endpoints included DCR at 12 weeks, objective response rate (ORR) at 6 and 12 weeks, progression-free-survival (PFS), overall survival (OS), and safety profile. Fifty-eight patients received cetuximab as monotherapy. The median age was 83.2 (range, 47.4 to 96.1). The majority of patients was chemotherapy naïve. The median follow-up was 11.7 months (95% CI: 9.6-30.1). The DCR at 6 and 12 weeks was 87% and 70%, respectively. The ORR was 53% and 42%, respectively, at 6 and 12 weeks. The median PFS and OS were 9.7 months (95% CI: 4.8-43.4) and 17.5 months (95% CI: 9.4-43.1), respectively. Fifty-one patients (88%) experienced toxicity, and 67 adverse events related to cetuximab occurred. Most of them (84%) were grade 1 to 2. Our study shows that cetuximab is safe and efficient for the treatment of patients, even elderly ones, with advanced cSCC. These results indicate that cetuximab is a promising agent to test in new combinations, especially with immune checkpoint inhibitors such as anti–PD-1 agents.

## INTRODUCTION

Nonmelanoma skin cancers, 20% of which are cutaneous squamous cell carcinomas (cSCC), are the most common malignant tumor in western countries. The incidence of cSCC is increasing yearly, and European data show that the age-standardized incidence ranges from 9 to 96 per 100,000 male inhabitants and 5 to 68 per 100,000 female inhabitants. The mean age at diagnosis is 74.4 years in males and 77.0 years in females [1–3]. In general, cure rates exceed 90% with early-stage disease. Conversely, the 5-year overall survival rate is below 50% for patients with local lymph node metastases and less than 10% for those with distant metastases [4–7]. The treatment of unresectable or metastatic cSCC remains highly challenging. Investigation of systemic therapies for advanced cSCC has been limited to a few prospective trials, and most of the time, retrospective data concern a highly selected population. Cisplatin-based combination chemotherapies are the most commonly used treatment, with an overall response rate of up to 80% [8–10]. Nevertheless, most of the time, this efficacy is not durable. Moreover, its use is often limited by a poor safety profile, with many adverse events, especially in elderly patients, who are the largest population of concern in the field of cSCC. Indeed, a large proportion of patients with unresectable cSCC are ineligible to receive standard chemotherapy regimens because of age (>70 years), Performance Status (PS) ≥1, or severe comorbidities (ie, cardiac or renal insufficiency).

Cetuximab has been tested in advanced cSCC and demonstrated a DCR of 69% at 6 weeks [11–14]. Additionally, there is a rationale based on preclinical data to block the programmed cell death protein 1 (PD-1) and/or the PD-1 ligand (PD-L1) pathway in inoperable cSCC. Recently, cemiplimab, a human monoclonal antibody directed against PD-1, showed response rates around 50% in this indication, with durable responses [15, 16]. It is very likely that this immunotherapy will quickly become the first-line treatment for advanced cSCC, and it will certainly be interesting to combine it with cetuximab. Before designing these new combination protocols, it is necessary to have additional clinical data on clinical outcomes of cetuximab. Therefore, in this retrospective, multicentre study, we evaluated the efficacy and safety of cetuximab as a single agent in patients with locally advanced or metastatic cSCC.

## RESULTS

### Patient characteristics

Between May 2007 and December 2017, a total of 58 patients (male, n=38; female, n=20) with advanced cSCC were treated with cetuximab as monotherapy and were enrolled in this study. The median age was 83.2 (range, 47.4 to 96.1) years. The most common primary site of the tumor was the head and neck (60.3%), followed by the extremities (27.6%) and the trunk (12.1%). Of the 58 patients, 19 (32.8%) were immunosuppressed (history of steroid use for more than 6 months [n= 7]; organ transplant [n= 2]; other solid cancer [n= 1]; failure of heart, lung, or kidney [n= 7]; or chronic lymphocytic/myeloid leukemia [n=2]).

Of these patients, 38 (65.5%) had unresectable local disease, 8 (13.8%) had regional lymph node involvement, and 12 (20.7%) had distant metastases. The main metastatic locations were the lung (n=6) and the skin (n=4), followed by the liver (n=1) and bone (n=1). No brain metastasis was documented.

More than 90% of the patients were chemotherapy naïve, and 3 (5.2%) had received previous radiotherapy alone (but at least one month before starting cetuximab). Twenty-one (36.2%) did not receive any treatment before starting cetuximab. Patients and tumor characteristics are summarized in Table 1.

**Table 1:** Baseline demographics of the patient population

Characteristic	No. of patients (%)
	(N = 58)
Median age, years [range]	83.2 years (47.4-96.1)
Sex	
Male	38 (65.5)
Female	20 (34.5)
ECOG-Performance status	
0	10 (17.2)
1	39 (67.2)
2	9 (15.6)
Immunosuppression	
Yes	19 (32.8)
No	39 (67.2)
Primary tumor location, No (%)	
Head and neck	35 (60.3)
Extremity	16 (27.6)
Trunk	7 (12.1)
Previous therapy	
None	21 (36.2)
Surgery alone	20 (34.5)
Radiotherapy alone	3 (5.2)
Surgery and radiotherapy	10 (17.2)
Surgery and radio-chemotherapy	4 (6.9)
AJCC, No. (%)	
Local	38 (65.5)
Lymph node	8 (13.8)
Distant metastases	12 (20.7)

Abbreviations: AJCC, American Joint Committee on Cancer; ECOG, Eastern Cooperative Oncology Group; PS, performance status.

### Exposure to cetuximab

The mean time between the previous treatment (corresponding most of the time to surgery for primary cSCC [54.1%] or surgery plus radiotherapy [27.0%]) and the first dose of cetuximab was 20.1 months (SD: 55.1). The mean duration of treatment was 4.2 months. The mean number of cetuximab infusions was 14 (SD: 12). Four patients (6.9%) received only one infusion, 23 patients (39.6%) received between 6 and 18 infusions, and 31 patients (53.5%) received more than 18 infusions. Most of the patients started to receive cetuximab weekly (77.6%); for the others (22.4%), the treatment was given every 2 weeks. Grade 3 to 4 cetuximab-related adverse events (AEs) led to discontinuation of cetuximab in nine patients. No dose reduction was performed.

### Cetuximab efficacy

Fifty-eight patients were included, and 55 were eligible for primary endpoint calculation (response at week 6); for three of them, evaluation according to the RECIST criteria was not available. Fifty patients were evaluated for the secondary endpoint (response at week 12). The median follow-up was 11.7 months (95% CI: 9.6-30.1). At week 6, the DCR was 87% (95% CI, 75.5% to 94.7%), and it was 70% (95% CI, 55.4% to 82.1%) at week 12. Of the 55 patients, 3 (5.5%) achieved a complete response (CR), 26 (47.2%) achieved a partial response (PR), 19 (34.6%) had stable disease, and 7 (12.7%) had progressive disease (PD). The ORR was 52.7% and 42%, respectively, at 6 and 12 weeks (Table 2). Table 3 summarizes the response to cetuximab therapy according to the stage of the disease. The efficacy of cetuximab has also been stratified according to several parameters and is summarized in Supplementary Table 1.

**Table 2:** Response and Disease Control Rates

Variable	Response at 6 weeks (n=55)			Response at 12 weeks (n=50)		
	No.	%	95% CI	No.	%	95% CI
**Complete response**	3	5.5%	[1.2-15.1]	1	2%	[0.05-10.6]
**Partial response**	26	47.2%	[33.7-61.2]	20	40%	[26.4-54.8]
**Stable disease**	19	34.6%	[22.2-48.6]	14	28%	[16.2-42.5]
**Progressive disease**	7	12.7%	[5.3-24.5]	15	30%	[17.9-44.6]
**Objective response rate**	29	52.7%	[38.8-66.3]	21	42%	[28.2-56.8]

**Table 3:** Response and Disease Control Rates according to stage of the disease

No. of Patients	All patients	Locally advanced	Regional disease	Metastatic disease
	(n=55-50)	(n=36-31)	(n=8-8)	(n=11-11)
**Complete response, week 6 (W6)-week 12 (W12)**	3-1	3-1	0-0	0-0
**Partial response, (W6-W12)**	26-20	15-11	6-4	5-5
**Stable disease, (W6-W12)**	19-14	13-9	2-3	4-2
**Progressive disease, (W6-W12)**	7-15	5-10	0-1	2-4

**Figure 1:**
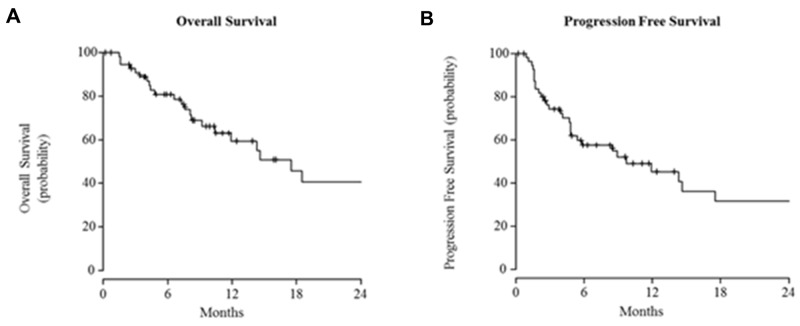
Kaplan-Meier curves for **(A)** overall survival **(B)** and progression-free survival.

The median PFS and OS were 9.7 months (95% CI: 4.8-43.4) and 17.5 months (95% CI: 9.4-43.1), respectively (Figure 1). At 18 months the survival rate was 46%, and it was 41% at 24 months.

### Cetuximab safety and tolerability

Fifty-one patients (88%) experienced toxicity, and 67 AEs related to cetuximab were reported. The majority of AEs observed were consistent with those previously reported in the literature, and most of them were grade 1 to 2 (84%). The most frequent toxicity was cutaneous (54%), and most of the time it was a typical cetuximab-induced folliculitis reaction, with 53% percent of the population (31 of 58) affected. Sixteen percent comprised serious AEs (grade 3-4) related to cetuximab, and involved 6 patients (10%). Cetuximab had to be discontinued in 1 patient because of an immediate hypersensitivity reaction after the first injection (grade 4). All other grade 3 AEs (n= 8) were related to folliculitis reactions, and 5 of them led to a transient discontinuation of cetuximab. No deaths related to treatment occurred. Major AEs are listed in Table 4.

**Table 4:** Most Common or Relevant Cetuximab-Related Adverse Event Categories by NCI CTC Toxicity Grade (n=58)

Adverse Event	All grades		Grade 3 to 4	
Category	No. of patients	%	No. of patients	%
Any category	51	88	6	10
Folliculitis reaction	31	53	8	0
Asthenia	13	22	0	0
Dry skin/pruritis	4	7	0	0
Nausea/vomiting	3	5	0	0
Nail/hand disorder	2	4	0	0
Diarrhea	2	4	0	0
Infusion-related reactions	2	4	1	2
Pilosity disorder	2	4	0	0
Pyrexia	2	4	0	0
Infection	1	2	0	0
Headache	1	2	0	0
Interstitial pneumonitis	1	2	0	0

Abbreviations: NCI CTC, National Cancer Institute Common Toxicity Criteria.

## DISCUSSION

To the best of our knowledge, this is the largest cohort reporting clinical outcomes of cetuximab prescribed in real-life conditions in unselected patients with locally advanced unresectable or metastatic cSCC. This study confirms its efficacy as a single agent, with an acceptable safety profile. In a large majority of chemotherapy-naive patients (93%), cetuximab can also be considered as first-line treatment, even in elderly patients (median age of 83.2 years). Furthermore, it is important to keep in mind that our cohort included a large proportion of immunocompromised patients (~33%), who are always excluded from clinical trials, and for whom the clinical outcomes with immunotherapy are more uncertain. For these reasons, frontline use of cetuximab also appears to be a good approach.

Our results reveal a higher efficacy compared with that previously reported, with an overall DCR and ORR of 87% and 53% at 6 weeks, respectively. Indeed, Maubec et al., in a phase II prospective trial including 36 chemotherapy-naïve patients with unresectable cSCC treated with cetuximab in the first line, reported a DCR of 69% and an ORR of 28% at 6 weeks [11]. The same proportion of patients achieved a DCR and 31% achieved an ORR with panitumumab, another monoclonal anti-*EGFR* antibody, in the phase II study conducted by Foote et al. [17]. We reported also, in another retrospective cohort of 31 patients, a DCR and ORR at week 6 of 68% and 48%, respectively [12]. Of course, due to the present study’s retrospective design, and because cross-study comparisons should be interpreted with care, our results have to be read with caution. One of the reasons that could explain our higher response rates is the fact that, in our cohort, ~66% of the population had local disease compared with 39% and 47% in the studies from Maubec et al. and Picard et al., respectively [11, 12]. Conversely, only ~14% of our patients had lymph node disease, while in the studies of Maubec et al. and Picard et al., 47% and 44% of enrolled patients had regional disease, respectively. It is difficult to compare these studies with the panitumumab study because the authors regrouped local and regional disease (81%).

The safety profile in our population was favorable and slightly better than in the other studies. Almost all of the patients had at least one AE (88%) compared with 100% in the studies by Maubec and Foote [11, 17]. The most frequent AE was, as expected, an inflammatory folliculitis reaction, occurring in 53% of the patients compared with 87% and 100% in previous studies. Sixteen percent of patients had serious AEs (grade 3-4) related to study treatment compared with 10% of the patients in Maubec’s study [17]. The higher percentage of serious AEs (31%) observed in the Australian study is largely due to the expected cetuximab-induced folliculitis. The authors suggest that the severity of this reaction is related to the fair skin of some Australians, who are exposed chronically and intensely to UV radiation [11]. It is also important to underline that Foote et al. used the terms “rash” and “dermatology” as AEs in their manuscript, perhaps overestimating the percentage of real acne-like rash.

Despite these data, it is important to keep in mind that the median PFS and median OS that we observed were only 9.7 and 17.5 months, respectively; these values were shorter in the study by Maubec et al. at 4.1 and 8.1 months, respectively [11]. This result highlights the importance of continuing additional clinical research. Very recently, it has been shown that cemiplimab (highly potent human monoclonal antibody directed against PD-1) is able to induce a response in approximately half of the patients. The estimated probabilities of PFS and OS at 12 months were 53% and 81%, respectively [15]. Longer-term survival data are needed, but investigation of cetuximab in combination with an anti–PD-1 agent could be relevant. A Phase II trial combining avelumab with or without cetuximab should be starting very soon (NCT03944941).

In conclusion, our study confirms the efficacy and acceptable tolerance of cetuximab as a single agent in first-line treatment of advanced cSCC. Definitively, cetuximab may be considered as a therapeutic option in this setting, particularly for elderly patients in whom chemotherapy is not appropriate. Several clinical trials have shown that anti–PD-1 agents are active in cSCC. Further clinical evaluations are needed to determine the role of cetuximab and immune checkpoint inhibitors in combination.

## MATERIALS AND METHODS

### Patient selection

We conducted a retrospective, multicentre study (13 French centres) from May 2007 to April 2017. The study was registered in the ClinicalTrials.gov protocol registration system (NCT03325738) and was conducted in accordance with the ethical principles of the Declaration of Helsinki and according to good clinical practice.

The eligibility criteria were: (1) patients 18 or older with histologically confirmed cSCC; (2) locally advanced and surgically unresectable cSCC or metastatic cSCC with documented progression; (3) cSCC treated with single-agent cetuximab; (4) Eastern Cooperative Oncology Group score ≤2; (5) presence of at least one measurable target lesion according to Response Evaluation Criteria in Solid Tumors (RECIST) criteria; (6) adequate hematologic, hepatic, and renal functions; (7) available medical data; (8) and affiliation of patient with French social security.

The criteria for unresectability were determined by a multidisciplinary committee composed of dermatologists, surgeons, and radiation therapists who evaluated the inability to achieve complete resection as well as the surgical impairment of critical cosmetic or functional outcomes. Patients with recurrent primary cSCC who had prior surgery, radiotherapy, and chemotherapy were eligible.

Patients were excluded if they met one of the following criteria: (1) prior therapy with an agent that targets EGFR; (2) prior radiotherapy within the last 4 weeks before the start of cetuximab; and (3) unstable systemic diseases or active uncontrolled infections.

### Objectives

The primary endpoint was the DCR at 6 weeks, defined as the percentage of patients who achieved a CR, PR, or stable disease at week 6.

The secondary endpoints were: (1) the DCR at 12 weeks; (2) the objective response rate (ORR), defined as the percentage of patients who achieved a CR or PR at weeks 6 and 12; (3) the PFS, defined as the delay between the first dose of cetuximab and the earliest day of progression or death, or the date of last follow-up in patients who were progression free and still alive at the end of the follow-up; and (4) the overall survival (OS), defined as the time between the first infusion of cetuximab and the last known patient update or the date of death; and (5) the safety profile. The type, frequency, severity, and time to onset of side effects were reported. Adverse events and grades were recorded according to National Cancer Institute Criteria, version 4.0.

### Treatment

Cetuximab was administered as an intravenous infusion. The standard schedule was an initial dose of 400mg/m^2^ followed by weekly 1-hour infusions of 250mg/m^2^. Cetuximab could be administered every 15 days according to the habits of the investigator. Patients received pretreatment with an oral antihistamine. The doses could be reduced at the beginning or during treatment according to the patient’s condition or toxicities. Cetuximab could be continued as long as the response or the stabilization persisted.

### Assessments

Response was evaluated every 6–8 weeks by repeated clinical and computed tomographic scan assessments on the basis of the extent of disease at presentation. Antitumor activity was evaluated according to the Response Evaluation Criteria in Solid Tumors 1.1 criteria [18].

### Statistical design

#### Analysis of qualitative data

Qualitative data, such as response rate, were presented as absolute frequency and relative frequency.

#### Analysis of quantitative data

Quantitative data were presented as mean, standard deviation, median and range.

Survival distributions were estimated by the Kaplan–Meier method.

## 





## Abbreviations

AEs: Adverse events
CR: Complete response
cSCC: Cutaneous squamous cell carcinoma
DCR: Disease control rate
ORR: Objective response rate
PD: Progressive disease
PFS: Progression-free-survival
PR: Partial response
OS: Overall survival
