# Supplementary material for: Cetuximab is efficient and safe in patients with advanced cutaneous squamous cell carcinoma: a retrospective, multicentre study
Source: Oncotarget. 2020 Jan 28;11(4):378–85. doi: 10.18632/oncotarget.27434 (PMC6996917; doi:10.18632/oncotarget.27434)
Supplement: Supplementary file 1 [file oncotarget-11-378-s001.pdf]

Cetuximab is efficient and safe in patients with advanced cutaneous squamous cell carcinoma: a retrospective, multicentre study

SUPPLEMENTARY MATERIALS TABLE

Supplementary Table 1: Response and Disease Control Rates based on stratification of patients.

See Supplementary File 1
