# Supplementary material for: Cetuximab is efficient and safe in patients with advanced cutaneous squamous cell carcinoma: a retrospective, multicentre study
Source: Oncotarget. 2020 Jan 28;11(4):378–85. doi: 10.18632/oncotarget.27434 (PMC6996917; doi:10.18632/oncotarget.27434)
Supplement: Supplementary file 2 [file oncotarget-11-378-s002.doc]

**Supplementary Table 1: Response and Disease Control Rates based on stratification of patients.**

| **Variable** | **Response at 6 weeks** | | | | | | |
| --- | --- | --- | --- | --- | --- | --- | --- |
|  | Previously untreated  (n=19)  No. % 95%  CI | Chemotherapy naïve  (n=51)  No. % 95%  CI | Prior radiotherapy treatment  (n=15)  No. % 95%  CI | Immunosuppressed  (n=19)  No. % 95%  CI | Not immunosuppressed  (n=36)  No. % 95%  CI | ≤18  perfusions of cetuxmiab  (n=37)  No. % 95%  CI | >18  perfusions of cetuximab  (n=12)  No. % 95%  CI |
| **Complete response** | 1 (5.3)  [0.1-26.0] | 2 (3.9)  [0.5-13.5] | 0 (0)  [0-21.8] | 0 (0.0)  [0.1-22.5] | 3 (8.3)  [1.8-22.5] | 2 (5.4)  [0.1-18.2] | 0 (0)  [0-26.5] |
| **Partial response** | 9 (47.4)  [24.4-71.13] | 25 (49)  [34.7-63.4] | 7 (46.7)  [21.3-73.4] | 13 (68.4)  [20.8-53.8] | 13 (36.1)  [20.8-53.8] | 18 (48.6)  [31.9-65.6] | 7 (58.3)  [27.7-84.8] |
| **Stable disease** | 7 (36.8)  [16.3-61.6] | 18 (35.3)  [22.4-49.9] | 4 (26.7)  [7.8-55.1] | 3 (15.8)  [3.4-39.6] | 16 (44.4)  [27.9-61.9] | 12 (32.4)  [18.0-49.8] | 5 (41.7)  [15.2-72.3] |
| **Progressive disease** | 2 (10.5)  [1.3-33.13] | 6 (11.8)  [4.4-23.9] | 4 (26.7)  [7.8-55.1] | 3 (15.8)  [3.4-39.6] | 4 (11.1)  [3.1-26.1] | 5 (13.5)  [4.5-28.8] | 0 (0)  [0-26.5] |
| **Objective response rate** | 10 (52.6)  [28.9-75.6] | 27 (52.9)  [38.5-67.1] | 7 (46.7)  [21.3-73.4] | 13 (68.4)  [43.4-87.4] | 16 (55.6)  [27.9-61.9] | 20 (54.1)  [36.9-70.5] | 7 (58.3)  [27.7-84.8] |

| **Variable** | **Response at 12 weeks** | | | | | | |
| --- | --- | --- | --- | --- | --- | --- | --- |
|  | Previously untreated  (n=18)  No. % 95% CI | Chemotherapy naïve  (n=47)  No. % 95% CI | Prior radiotherapy treatment  (n=11)  No. % 95% CI | Immunosuppressed  (n=16)  No. % 95%  CI | Not Immunosuppressed  (n=34)  No. % 95%  CI | ≤18 perfusions of cetuxmiab  (n=31)  No. % 95%  CI | >18 perfusions of cetuximab  (n=12)  No. % 95%  CI |
| **Complete response** | 0 (0)  [0.0-18.5] | 0 (0)  [0-7.5] | 0 (0)  [0-28.5] | 0 (0.0)  [0.0-20.6] | 1 (2.9)  [0.0-15.3] | 1 (3.0)  [0.0-15.7] | 0 (0.0)  [0.0-26.5] |
| **Partial response** | 7 (38.9)  [17.9-64.3] | 19 (40.4)  [26.4-55.7] | 4 (36.4)  [10.9-69.2] | 7 (43.8)  [19.8-70.1] | 13 (38.2)  [22.2-56.4] | 11 (39.4)  [22.9-57.9] | 6 (50.0)  [21.1-78.9] |
| **Stable disease** | 3 (16.7)  [35.8-41.4] | 13 (27.7)  [15.6-42.6] | 4 (36.4)  [10.9-69.2] | 5 (31.2)  [11.0-58.7] | 9 (26.5)  [12.9-44.4] | 7 (21.2)  [9.0-38.9] | 6 (50.0)  [21.1-78.9] |
| **Progressive disease** | 8 (44.4)  [21.5-69.2] | 15 (31.9)  [19.1-47.1] | 3 (27.3)  [6.0-61.0] | 4 (25.0)  [0.7-53.4] | 11 (32.4) [17.4-50.5] | 12 (36.4)  [20.4-54.9] | 0 (0.0)  [0.0-26.5] |
| **Objective response rate** | 7 (38.9)  [17.3-64.3] | 19 (40.4)  [26.4-55.7] | 4 (36.4)  [10.9-69.2] | 7 (43.8)  [19.8-70.1] | 14 (41.2)  [26.6-59.3] | 12 (38.7)  [25.5-60.8] | 6 (50)  [21.1-78.9] |
